# The association between visual impairment and fatigue: a systematic review and meta‐analysis of observational studies

**DOI:** 10.1111/opo.12647

**Published:** 2019-11-06

**Authors:** Wouter Schakel, Christina Bode, Ellen B M Elsman, Hilde P A van der Aa, Ralph de Vries, Gerardus H M B van Rens, Ruth M A van Nispen

**Affiliations:** ^1^ Department of Ophthalmology Amsterdam University Medical Centers Vrije Universiteit Amsterdam The Netherlands; ^2^ Amsterdam Public Health Research Institute Amsterdam The Netherlands; ^3^ Department of Psychology, Health and Technology University of Twente Enschede The Netherlands; ^4^ Medical Library Vrije Universiteit Amsterdam The Netherlands; ^5^ Department of Ophthalmology Elkerliek Hospital Helmond The Netherlands

**Keywords:** fatigue, meta‐analysis, quality of life, systematic review, vision disorders, visually impaired persons

## Abstract

**Purpose:**

The aim was to compare fatigue levels between patients with visual impairment and controls with normal sight and to examine the association between fatigue and vision loss severity.

**Methods:**

A systematic literature search was performed using databases of PubMed, Embase, PsycINFO and Cochrane to identify observational studies with outcomes related to fatigue (e.g. vitality subscale of the Short‐Form 36, Fatigue Assessment Scale). A meta‐analysis was performed using standardised mean differences (SMDs) and odds ratios (OR) to quantitatively summarise the association between visual impairment and fatigue. Sources of heterogeneity were explored by subgroup and sensitivity analyses. Study quality was assessed with the Newcastle‐Ottawa scale.

**Results:**

After reviewing 4477 studies, 22 studies with a total of 40 004 participants were included, of which 18 contributed to meta‐analysis. Among these, eight were assessed as moderate quality studies and 10 as high quality studies. Pooled analysis involving 2500 patients and 8395 controls showed higher fatigue severity levels (S.M.D. = −0.36, 95% CI −0.50 to −0.22, 14 studies) among visually impaired patients compared to normally sighted controls. This effect size was small and persisted in sensitivity analyses that involved study quality, fatigue assessment tools and visual acuity data. Furthermore, pooled analysis of four studies including 2615 patients and 5438 controls showed a significant association between visual impairment and fatigue (OR = 2.61, 95% CI 1.69 to 4.04). Secondary meta‐analysis of four studies showed no significant difference in fatigue severity (S.M.D. = 0.01, 95% CI −0.37 to 0.39) between patients with moderate visual impairment and patients with severe visual impairment or blindness.

**Conclusions:**

Current moderate to high quality evidence suggest that patients with visual impairment experience more severe fatigue symptoms than persons with normal sight. However, a limited number of available studies indicates that fatigue is not associated with severity of vision loss. Future studies are required to determine which factors and underlying mechanisms may explain the association between visual impairment and fatigue. Discussing fatigue at an early stage and developing intervention options for vision‐related fatigue should be considered within the field of low vision rehabilitation.

## Introduction

Visual impairment and blindness are highly prevalent conditions in the Western world that are primarily caused by age‐related eye conditions. Globally, the number of persons affected by moderate to severe visual impairment and blindness is estimated to increase from 253 million in 2015 to approximately 276 million in 2020 due to growing and aging populations.^1^ Permanent vision loss is often caused by chronic eye disorders that slowly progress in severity over time, such as age‐related macular degeneration (AMD) or glaucoma, and can therefore assert a detrimental effect on a patient's mental health^2^, ^3^ and quality of life.^4^ In addition to the individual burden, visual impairment and blindness have also been recognised as a cause of considerable economic burden to society at large.^5^

More recently fatigue has been suggested as an important problem for persons with visual impairment that does not seem to improve by general low vision services.^6^ Patients with various causes of visual impairment have described fatigue as an overwhelming sensation of tiredness with mental and physical manifestations.^7^ In our previous study, we found that adults with visual impairment experienced higher levels of fatigue and were four times more likely to experience severe impact of fatigue on daily life compared to adults with normal sight.^8^ This may be because persons with vision loss require more effort to establish visual perception, have to invest more cognitive resources for practical adjustments in daily life, experience difficulties under suboptimal lighting conditions, or struggle with negative cognitions or depressed mood.^7^ Even though some studies indicate an association between fatigue and severity of vision loss,^9^, ^10^ there seem to be a limited number of studies that address fatigue as a primary research outcome in this population, and, to the best of our knowledge, results have not yet been synthesised. Consequently, the magnitude of fatigue severity in patients with visual impairment is still not fully understood.

Some indications about the impact of fatigue in visually impaired people (in comparison with normally sighted people) can be found in studies on quality of life of people with visual impairment. These studies apply generic quality of life questionnaires to compare different target groups. Regarding the construct of fatigue, a commonly used instrument is the Medical Outcomes Study Short‐Form 36 questionnaire (SF‐36)^11^ that measures ‘vitality’ as a separate domain of health‐related quality of life. This subscale was developed to measure bidirectional concepts of energy and fatigue, with higher scores being indicative of ‘full of energy’ and lower scores representing ‘feeling tired and worn out’.^11^ Several observational studies have incorporated the SF‐36 to quantify the impact of visual impairment on health‐related quality of life. A systematic inventory of these outcomes as a proxy for fatigue may enable us to more reliably evaluate the association between fatigue severity and visual impairment. Therefore, the goal of this study was to perform a meta‐analysis of observational studies (1) to compare fatigue levels between visually impaired patients and normally sighted controls, and (2) to examine the association between fatigue and severity of vision loss.

## Methods

A review protocol was developed based on the Preferred Reporting Items for Systematic Reviews and Meta‐Analysis (PRISMA)‐statement.^12^ The meta‐analysis was conducted and reported in accordance with the Meta‐analysis Of Observational Studies in Epidemiology (MOOSE) guidelines.^13^

### Search method and selection procedure

A comprehensive search was performed in the bibliographic databases PubMed, Embase.com, Ebsco/PsycINFO and Wiley/Cochrane Library in collaboration with a medical librarian. Databases were searched from their date of inception up to 3 April 2019. Terms (including synonyms and closely related words) related to visual impairment, blindness, eye conditions, fatigue and quality of life were used as index terms or free‐text words. In Embase.com a limitation was added for ‘Quality of Life’. The search was performed without date, language, conference abstract or publication status restriction. Duplicate articles were excluded. The full search strategies for all databases can be found in the Supplementary Information (*Appendix* S1). Two researchers independently reviewed articles on title and abstract against the inclusion criteria using Rayyan software (rayyan.qcri.org),^14^ a web‐based application designed for systematic reviews. Potential articles that met the criteria were subsequently reviewed on full text for eligibility. Discrepancies were resolved by discussion or by consultation of a third researcher where necessary.

### Eligibility criteria

The following inclusion criteria were used: (1) original research reported or accessible in English; (2) studies with a cross‐sectional or experimental design; (3) participants with at least moderate visual impairment according to the World Health Organization (WHO) criteria, defined as presenting visual acuity (VA) worse than 20/60 (6/18, 0.33) and/or visual field worse than 30 degrees in the better‐seeing eye,^15^ or on the basis of similar information or other indications of severe vision loss; (4) participants aged ≥ 18 years; (5) data on fatigue severity or the prevalence of fatigue assessed by generic measures; (6) fatigue outcomes compared to normally sighted controls and/or fatigue comparisons between patients with different levels of visual impairment according to VA. To increase the certainty that fatigue was related to vision loss, studies were excluded if visual impairment was accompanied by the following chronic (inflammatory) conditions that are characterized by fatigue: Grave's ophthalmopathy, Behçet syndrome, uveitis or multiple sclerosis. Studies that used SF‐36 norm scores or previously reported data as reference groups were excluded from meta‐analysis but were summarised in a narrative review.

### Data extraction

The following characteristics of the studies were extracted: (1) country and year of publication; (2) study design; (3) sample information (cause of visual impairment, mean VA, age, gender distribution, sample size); (4) control condition; (5) fatigue outcome measure; (6) mean fatigue scores with standard deviations and/or the prevalence of fatigue based on the included outcome measure. In some instances standard deviations were calculated from the standard error (S.E.), sample size and 95% confidence interval (CI) as described in the Handbook of the Cochrane Collaboration.^16^ Corresponding authors were contacted by email to provide additional study data if parameters of interest were missing or not fully reported in the article (i.e. request for vitality means in studies reporting SF‐36 component scores). When data was reported for multiple groups, estimates were combined to facilitate fatigue comparisons exclusively between cases with visual impairment and controls with normal sight, as defined by our inclusion criteria.

### Quality assessment

As recommended by the Cochrane Collaboration,^17^ the methodological quality of all studies selected for meta‐analysis were assessed independently by two researchers using modified versions of the Newcastle–Ottawa Scale (NOS).^18^ For cross‐sectional studies, the adapted version by Herzog *et al*. (2013)^19^ was utilised, while for case‐control studies some modifications were made to the original NOS based on methods described in previous reviews.^20^ Both forms use a star rating system for quality assessment of three main parameters: selection and definition of study groups (0–4 stars); comparability of study groups (0–2 stars); and outcome assessment and/or soundness of statistical analysis (0–3 stars). The star ranking method was based on previous reviews.^21^ Summed NOS scores of ≤4 were ranked as poor quality studies, scores between 5–6 as moderate quality studies, and scores ≥ 7 as high quality studies.

### Statistical analysis

All meta‐analyses were performed with Cochrane Review Manager (RevMan) software version 5.3.5^22^ using the inverse variance method. Heterogeneity was determined prior to meta‐analysis using the I^2^ test, with values greater than 25%, 50% and 75% being indicative of low, moderate and high heterogeneity, respectively. Data was pooled with a random‐effects model in case of substantial heterogeneity (I^2^ > 50%), while a fixed‐effects model was applied for lower levels of heterogeneity.

For the first research question, separate meta‐analyses were performed to compare fatigue severity and the prevalence of fatigue between visually impaired patients and normally sighted controls. SF‐36 vitality scores were initially pooled to estimate the mean difference for fatigue severity. This subscale consists of four items that are transformed to a 0–100 summary score, where 0 represents the worst health state (‘feeling tired and worn out’) and 100 the most optimal one (‘feeling full of energy’). Standardised mean differences (S.M.D.) together with 95% confidence intervals were subsequently calculated to enable comparisons between different measures that were used for continuous fatigue outcomes. Pooled effect sizes for SMDs were defined by Hedges adjusted *g*, where 0.20 represents a small effect, 0.50 a medium effect, and ≥0.80 a large effect. We considered a S.M.D. of ≥0.5 as an important difference.^23^ Odds ratios (OR) and 95% CIs were determined for dichotomous variables to evaluate the association between visual impairment and the presence of fatigue. These estimates were pooled by meta‐analysis using the Mantel‐Haenszel odds ratio method. When studies reported both unadjusted and adjusted effect estimates, we selected the OR from the model that was adjusted for the maximum number of covariates.

Secondary meta‐analyses were performed to examine the association between fatigue severity and the degree of vision loss. Visual impairment comparison groups were selected by an exploratory approach. Studies were initially selected on the basis of stratified fatigue outcomes for various degrees of vision loss, and were subsequently compared on VA cut‐off scores used for categorisation of visual impairment groups. After a close inspection of the identified studies, we decided to compare fatigue severity between patients with moderate visual impairment and patients with severe visual impairment or blindness. In accordance with criteria of the WHO, moderate visual impairment was defined as VA worse than 20/60 (6/18, 0.33) and equal or better than 20/200 (6/60, 0.10), and severe visual impairment or blindness as VA worse than 20/200 (6/60, 0.10).^15^

Sources of heterogeneity were explored by subgroup comparisons when at least 10 studies were synthesised by meta‐analysis. The following subgroups were considered in the review protocol: overall study quality; cause of visual impairment (AMD, other specific eye disorders, other causes of visual impairment); study design (case‐control, cross‐sectional); vision loss severity (VA worse than 20/40 [6/12, 0.50], VA worse than 20/60 [6/18, 0.33], unknown VA); studied region (Asia, North America, Australia, Europe, South America); population (Western, Non‐Western); gender (≥60% female, even gender distribution, ≥60% male, unknown); diagnosis of visual impairment (ophthalmic evaluation, self‐report, record‐linkage). Sensitivity analyses were performed by excluding outlier studies in a step‐wise procedure and by removing all studies that failed to report VA outcomes for the ophthalmic sample.

## Results

### Search results

**Figure 1:**
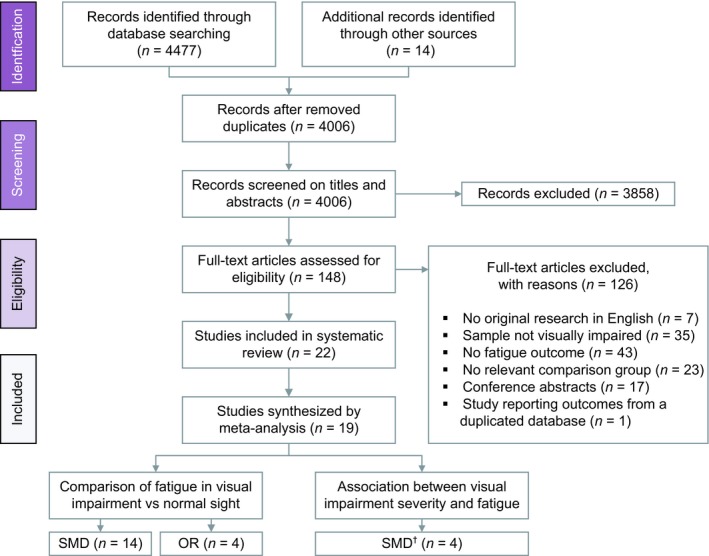
Flow‐diagram displaying the selection process for studies included in this meta‐analysis. S.M.D., standardised mean difference; OR, odds ratio, ^†^S.M.D. for moderate visual impairment and severe visual impairment/ blindness.

The database searches initially identified 4477 hits, of which 3992 articles were screened on titles and abstracts after duplicate removal (*Figure* 1). Among these, a total of 134 references remained for which full text versions were reviewed for the inclusion criteria. Agreement between the two reviewers was 97.7% for title and abstract screening and 79.3% for full text review. Together with 14 articles identified through manual searches and reference lists, this resulted in 22 articles that were included in this review. We received responses from six out of 19 contacted authors and two authors provided additional data.^24^, ^25^ Forty‐three articles were excluded because they had no fatigue outcomes; 35 articles because the participants were not visually impaired; 23 articles because they had no control group or used comparison groups that were not relevant for our analysis (e.g. comparison groups based on disease severity rather than presenting or post‐refraction VA); 17 articles because they provided insufficient data for meta‐analysis (e.g. conference abstract); seven articles because of language‐restrictions; and one article because they utilised the same sample source from a study that was already included.^10^ Of the 22 included studies, nine used a cross‐sectional design,^9^, ^10^, ^26^, ^27^, ^28^, ^29^, ^30^, ^31^, ^32^ eight compared cases with controls,^8^, ^24^, ^33^, ^34^, ^35^, ^36^, ^37^, ^38^ and five studies compared the target population to normative data.^25^, ^39^, ^40^, ^41^, ^42^ Findings from the normative comparison studies were summarised by a narrative approach. Finally, 19 studies provided sufficient data for quantitative synthesis by meta‐analysis.

### Characteristics of included studies

*Table* 1 describes the characteristics of the 22 articles included in this review. Altogether, the studies included a total of 40 004 participants with sample sizes ranging from 22^39^ to 22 486^9^ participants. They were published between 1998 and 2018; 13 of the 22 studies were carried out in the past 10 years. Six studies were conducted in North America,^28^, ^29^, ^33^, ^36^, ^40^, ^42^ six in Asia,^24^, ^27^, ^30^, ^31^, ^38^, ^39^ six in Europe,^8^, ^9^, ^25^, ^32^, ^34^, ^41^ two in Australia,^10^, ^37^ and two in Brazil.^26^, ^35^ With regard to participants with visual impairment, 9%^39^ to 82%^27^ were female and mean age ranged from 21^25^ to 87 years.^32^ In the majority of the studies, participants had visual impairment or blindness caused by various eye conditions^8^, ^9^, ^10^, ^24^, ^25^, ^26^, ^27^, ^28^, ^30^, ^31^, ^32^, ^36^, ^39^, ^40^. In contrast, two studies specifically included patients with glaucoma,^33^, ^35^ four studies solely focused on AMD,^29^, ^34^, ^37^, ^42^ and in two separate studies patients either had diabetic retinopathy (DR)^38^ or Usher syndrome type 1.^41^ Two studies specifically included patients who were legally blind^36^, ^39^ and one study included patients with multiple sensory impairments.^32^ Presence of visual impairment and/or diagnosis of ocular disease was determined by a comprehensive ophthalmologic examination (measurement of presenting and/or post‐refraction VA) in 10 studies,^10^, ^26^, ^27^, ^28^, ^29^, ^30^, ^31^, ^34^, ^40^ by examination of medical records in seven studies,^8^, ^25^, ^33^, ^35^, ^37^, ^38^, ^41^ by self‐reported measures in two studies,^9^, ^32^ and based on certificates for governmental disability benefits in three studies.^24^, ^36^, ^39^ With regard to fatigue outcomes, visual impairment was defined as best‐eye presenting VA worse than 20/60 (6/18, 0.33) in three studies;^8^, ^26^, ^27^ as best‐eye post‐refraction VA less than 20/60 (6/18, 0.33) in one study^30^; as best‐eye post‐refraction VA worse than 20/40 (6/12, 0.50) in one study^10^; as best‐eye presenting VA worse than 20/40 (6/12, 0.50) in one study^31^; as binocular presenting VA of 20/40 (6/12, 0.50) or worse in one study^28^; and as best‐eye post‐refraction VA of 20/1000 (6/300, 0.02) or worse in another study.^24^ Because of the limited number of identified studies, we decided to include all abovementioned criteria of visual impairment and blindness. For case‐control studies, control groups were comprised of persons without ocular disease in three studies,^34^, ^35^, ^37^ healthy participants without chronic disability in two studies,^36^, ^38^ persons with normal (self‐reported) vision in two studies,^8^, ^24^ and hospital controls without glaucoma in one study.^33^

**Table 1:** Characteristics of reviewed studies in alphabetic order on first author, divided into: (1) case‐control studies, (2) cross‐sectional studies, and (3) normative comparison studies

Study	Year	Country	Used in analysis	Fatigue measure	Visual impairment					Control subjects				
					*n*	VI cause, mean VA / VI definition	M age (years)	Gender %♀	M fatigue ± S.D. or % fatigue	*n*	Control condition	M age (years)	Gender %♀	M fatigue ± S.D. or % fatigue
1. Case‐control studies (*n* = 8)														
Cabrera^33^	2018	Canada	M2	STOP‐Bang: tiredness	437	Glaucoma	71	53%	48% fatigue	441	No glaucoma	69	53%	39% fatigue
Chatziralli^34^	2017	Greece	M1	SF‐36: vitality	114	AMD, monocular post‐refraction (unspecified) VA = 20/40 (6/12, 0.50)	77	45%	76 ± 11	100	No ocular disease	76	52%	85 ± 14
Cypel^35^	2004	Brazil	M1	SF‐36: vitality	102	Glaucoma, best‐eye post‐refraction (unspecified) VA = 20/60 (6/18, 0.33)	69	49%	71 ± 24	58	No ocular disease	63	66%	72 ± 21
Horner‐Johnson^36^	2010	USA	M1	SF‐36: vitality	25	Legal blindness: best‐eye presenting VA of 20/200 (6/60, 0.10) or worse	53	52%	55 ± 9	35	No chronic disability	40	61%	51 ± 12
Mathew^37^	2011	Australia	M1	SF‐36: vitality	145	AMD	78	63%	53 ± 20	104	No ocular disease	78	70%	55 ± 23
Schakel^8^	2018	NL	M1, M3	FAS MFIS	224	VI: best‐eye presenting VA of 20/66 (6/20, 0.30) or worse	57	64%	23 ± 6	233	No VI	45	73%	18 ± 5
									31 ± 17					20 ± 13
Tamura^24^	2014	Japan	M1	SF‐8: vitality	598	VI: best‐eye post‐refraction VA of 20/1000 (6/300, 0.02) or worse	60	38%	50 ± 7	615	No VI	56	62%	51 ± 6
Yu^38^	2013	China	M1	SF‐36: vitality	108	DR	‐	‐	56 ± 18	108	No VI and no chronic illness	‐	‐	63 ± 14
2. Cross‐sectional studies (*n* = 9)														
Chia^10^	2004	Australia	M1	SF‐36: vitality	66	VI: best‐eye post‐refraction VA worse than 20/40 (6/12, 0.50)	79	70%	51 ± 23	2916	no VI	66	57%	62 ± 22
Cypel^26^	2017	Brazil	M1, M3	SF‐36: vitality	77	VI: best‐eye presenting VA worse than 20/60 (6/18, 0.33)	†80‐100	†69%	56 ± 19	73	no/mild VI	†80‐100	†69%	62 ± 19
Dev^27^	2014	Nepal	M1	SF‐36: vitality	197	VI: best‐eye presenting VA worse than 20/60 (6/18, 0.33)	†75	82%	46 ± 12	75	no VI	†75	69%	48 ± 09
Fischer^28^	2009	USA	M1	SF‐36: vitality	401	VI: binocular presenting VA of 20/40 (6/12, 0.50) or worse, or contrast sensitivity worse than 1.55	‡71	‡51%	56 ± 21	1453	no VI	63	67%	64 ± 20
Knudtson^29^	2005	USA	M1	SF‐36: vitality	179	AMD (both eyes affected)	76	60%	56 ± 21	1356	no AMD	64	57%	66 ± 19
Kuang^30^	2005	Taiwan	M1	SF‐36: vitality	7	VI: best‐eye post‐refraction VA (pinhole corrected) worse than 20/60 (6/18, 0.33)	‐	‐	79 ± 14	166	no VI	‐	‐	86 ± 8
Mojon‐Azzi^9^	2008	Germany	M2	Fatigue (yes)§	679	Poor self‐reported eyesight	73	†56%	59% fatigue	3309	Excellent eyesight	61	†56%	23% fatigue
Tsai^31^	2004	Taiwan	M1	SF‐36: vitality	257	VI: best‐eye presenting VA worse than 20/40 (6/12, 0.50)	†72	†40%	68	1104	no VI	†72	†40%	73
Yamada^32^	2014	Czech Republic	M2	Fatigue (yes)¶	1275	VI: interRAI LTCF vision and hearing score ≥ 1	87	76%	37% fatigue	1455	no VI/HI	80	69%	18% fatigue
3. Normative comparison studies (*n* = 5)														
Elsman^25^	2018	NL	M3, NR	SF‐36: vitality	172	VI, 56% of patients had best‐eye presenting VA of 20/66 (6/20, 0.30) or worse	21	54%	59 ± 20	‐	NL norms	16‐40	♂♀	71 ± 16
Masaki^39^	2015	Japan	NR	SF‐36: vitality	11	Blindness: binocular post‐refraction VA of 20/2000 (6/600, 0.01) or worse	22	9%	42 ± 7	‐	JP norms	20‐29	♂	51 ± 10
Scott^40^	1999	USA	NR	SF‐36: vitality	156	VI, median best‐eye presenting VA = 20/200 (6/60, 0.10)	73	55%	65 ± 8	264	US norms	>75	♂♀	50 ± 24
Wahlqvist^41^	2016	Sweden	NR	HET: fatigue	60	Usher T1, best‐eye post refraction (unspecified) VA = 20/40 (6/12, 0.50)††	49	60%	62% fatigue	5738	SW norms	49	56%	49% fatigue
Williams^42^	1998	USA	M3, NR	POMS: fatigue	86	AMD, best‐eye presenting VA = 20/320 (6/95, 0.06)	79	51%	9 ± 5	505	US norms	83	♂♀	7 ± 6

AMD, age‐related macular degeneration; DR, diabetic retinopathy; FAS, Fatigue Assessment Scale; HET, Health on Equal Terms questionnaire; HI, hearing impairment; interRAI LTCF, The interRAI Long‐Term Care Facilities Assessment System; JP, Japan; M, mean; M1 meta‐analysis 1: S.M.D. of fatigue severity between visual impairment and normal sight; M2 meta‐analysis 2: OR of the presence of fatigue between visual impairment and normal sight; M3 meta‐analysis 3: S.M.D. of fatigue severity between moderate visual impairment and severe visual impairment or blindness; MFIS, Modified Fatigue Impact Scale; NL, the Netherlands; NR, narrative review; POMS, Profile Of Mood States; S.D., standard deviation; SF‐36, Medical Outcomes Study Short‐Form 36 questionnaire; STOP‐Bang snoring, tiredness, observed apnea, high BP, BMI, age, neck circumference, and male gender questionnaire; USA, United States of America; VA, visual acuity; VI, visual impairment. ♂♀, Males and females; ♂, Only males.

^†^ Estimate for all included participants;

^‡^ Estimate for participants with either hearing, vision or olfaction impairment;

^§^ Fatigue responding yes: undefined;

^¶^ Fatigue defined as unable to start/finish daily activities because of energy loss;

^††^ VI defined by concentric central field loss with a remaining peripheral island;

### Quality assessment

*Table* 2 shows the methodological quality of the individual studies pooled by meta‐analysis in this review. Overall, total scores ranged between 5 and 8 of a possible 9 points, indicating that the studies were of moderate to high quality. The percentage of high quality studies was 50% for case‐control^8^, ^24^, ^34^, ^37^ and 60% for cross‐sectional studies.^10^, ^25^, ^28^, ^29^, ^30^, ^31^ For both study designs, quality was predominately limited by unsatisfactory participation rates and poor or unknown comparability between responders and non‐responders. Other important sources of weaker quality were failure to control for confounding effects in principal analyses and the use of non‐validated measurement tools.

**Table 2:** Quality assessment based on the (modified) NOS

Study	Newcastle Ottawa Scale	Total score
	Selection/ Comparability/ Exposure	
Case‐control studies†		
Cabrera 2018^33^	★★☆★/ ☆☆/ ★★☆	5
Chatziralli 2017^34^	★★★★/ ★★/ ★★☆	8
Cypel 2004^35^	★★★★/ ☆☆/ ★★☆	6
Horner‐Johnson 2010^36^	☆★☆★/ ★★/ ★★☆	6
Mathew 2011^37^	★★★☆/ ★★/ ★★☆	7
Schakel 2018^8^	★★★☆/ ★★/ ★★☆	7
Tamura 2014^24^	★★★☆/ ★★/ ★★★	8
Yu 2013^38^	★★★☆/ ★★/ ★☆☆	6
Cross‐sectional studies‡		
Chia 2004^10^	★★☆★/ ★★/ ☆★★	7
Cypel 2017^26^	★★☆★/ ☆☆/ ☆★★	5
Dev 2004^27^	★★☆★/ ☆☆/ ☆★★	5
Elsman 2018^25^	★★☆★/ ★★/ ☆★★	7
Fischer 2009^28^	★★☆★/ ★★/ ☆★★	7
Knudtson 2005^29^	★★☆★/ ★★/ ☆★★	7
Kuang 2005^30^	★★☆★/ ★★/ ☆★★	7
Mojon‐Azzi 2008^9^	★★☆☆/ ★★/ ☆★★	6
Tsai 2004^31^	★★☆★/ ★★/ ☆★★	7
Yamada 2014^32^	★★☆☆/ ★★/ ☆☆★	5

^†^ Stars: positive (black) or negative (white) assessment of 1 = case definition; 2 = case representativeness; 3 = control selection; 4 = control definition; 5–6 = comparability; 7–8 = ascertainment methods; 9 = non‐response rate.

^‡^ Stars: positive (black) or negative (white) assessment of 1 = sample representativeness; 2 = sample size; 3 = non‐respondents; 4 = exposure ascertainment; 5–6 = comparability; 7–8; outcome assessment; 9 = statistical test.

### Normative comparisons

A total of five studies compared fatigue outcomes of persons with visual impairment to general populations or previously reported norm scores (*Table* 1). Scott *et al*. (1999) found that SF‐36 vitality scores of patients with low vision (mean = 64.7 ± 8.7) were significantly higher compared to an age‐matched population of the United States of America (mean = 50.41 ± 24.4), indicating that they experience less fatigue.^40^ In contrast, Elsman *et al*. (2019) found significantly worse vitality scores for young adults with visual impairment (mean = 59.2 ± 19.5) compared to age‐matched norm scores of the Dutch population (mean = 70.7 ± 16.4).^25^ Likewise, Masaki (2015) reported significantly lower vitality scores for young males with blindness (mean = 41.9 ± 7.2) compared to Japanese norm scores for young males aged between 20 and 29 years old (mean = 50.5 ± 10.2).^39^ Utilising the Profile of Mood States, William *et al*. (1998) reported higher fatigue severity for elderly patients with AMD (mean = 8.8 ± 5.1) relative to previously reported scores of community controls of similar age (mean = 6.7 ± 6.4).^42^ Using the Health on Equal Terms questionnaire, Wahlqvist *et al*. (2016) found that the odds for fatigue were 1.6 greater among patients with Usher syndrome 1 compared to Swedish population norms, which was significant.^41^ Taken together, despite the different fatigue measures and study populations included, these findings seem to indicate that patients with visual impairment experience increased fatigue levels compared to persons with normal vision.

### Meta‐analysis

#### Visual impairment vs normal sight: fatigue severity

**Figure 2:**
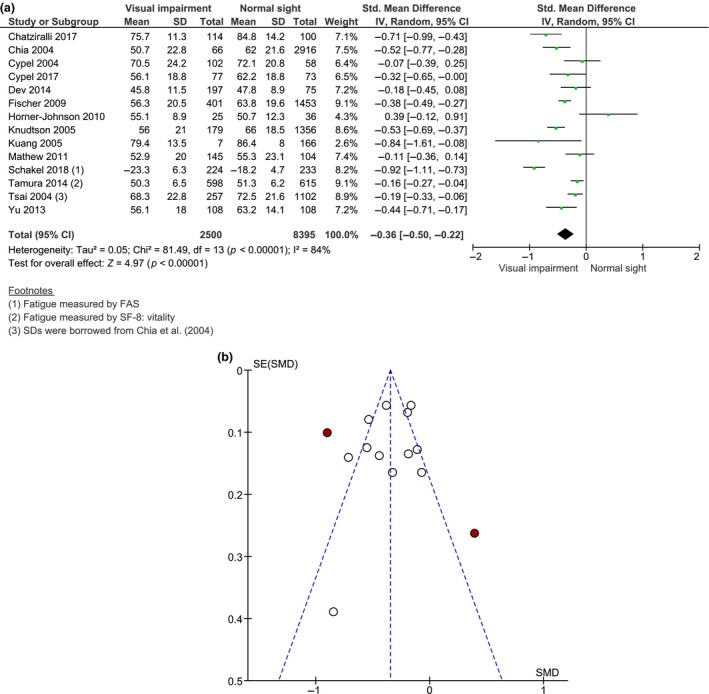
(a) Forest plot of meta‐analysis: S.M.D. of fatigue severity between visually impaired patients and normally sighted controls (*n* = 14). S.M.D., standardised mean difference; S.D., standard deviation; CI, confidence interval; FAS, Fatigue Assessment Scale; SF‐8 Medical Outcomes Study Short‐Form 8 questionnaire. (b) Funnel plot for assessment of publication bias among studies included in the meta‐analysis comparing fatigue severity between visually impaired patients and normally sighted controls. S.E., standard error; S.M.D., standardised mean difference. Red circles represent individual studies that were considered as outliers.

For our primary aim, we identified 14 studies comparing fatigue severity levels of visually impaired patients to those of normally sighted controls. The SF‐36 vitality subscale was used in 13 studies^10^, ^24^, ^26^, ^27^, ^28^, ^29^, ^30^, ^31^, ^34^, ^35^, ^36^, ^37^, ^38^ and the Fatigue Assessment Scale (FAS) was used in one study^8^ to measure fatigue severity. Random effects models were chosen because of substantial levels of heterogeneity between studies (*I* ^2^ = 84%–87%). A significant pooled mean difference was found for vitality scores (MD = −5.03, 95% CI −7.50 to −2.55, *n* = 13), suggesting that visually impaired patients experience higher levels of fatigue compared to control subjects with normal sight. *Figure* 2a shows the meta‐analysed results for all continuous fatigue measures based on the fourteen studies mentioned above, comparing 2475 cases and 8395 controls. There were considerably more controls than cases due to low visual impairment prevalence rates found in three large population‐based cross‐sectional studies (see *Table* 1).^10^, ^28^, ^29^ The forest plot revealed a significant pooled S.M.D. (−0.36, 95% CI −0.50 to −0.22, *I* ^2^ = 84%, *Figure* 2a), indicating that visually impaired patients had more severe fatigue symptoms than normally sighted controls. Visual inspection of the funnel plot suggested no asymmetry except for two outlier studies (highlighted in red),^8^, ^36^ indicating possible publication bias (*Figure* 2b). Sensitivity analyses revealed a reduction of heterogeneity (from 84% to 73%) after removing the study by Schakel *et al*. (2018),^8^ which measured fatigue severity with the FAS instead of SF‐36 vitality and had a clearly larger difference in average fatigue between the groups. Furthermore, heterogeneity was even further reduced to 64% by excluding five studies^24^, ^29^, ^36^, ^37^, ^38^ with missing VA values for the ophthalmic patients under investigation. The statistical significance and the magnitude of the pooled effect persisted for the remaining eight studies (S.M.D. = −0.30, 95% CI −0.43 to −0.18, *I* ^2^ = 64%).

Exploratory Subgroup analyses (*Table* 3) revealed that studied region and gender were moderating variables for the pooled S.M.D. of fatigue severity, meaning that the effect of visual impairment varied per region and with different types of gender distributions. Studies conducted in South America, Asia and Europe were characterised by low heterogeneity compared to substantial heterogeneity for studies performed in North America and Australia (*Appendix* S2). Furthermore, European studies showed the highest pooled S.M.D. within regional comparisons (S.M.D. = −0.84, 95% CI −1.04 to −0.64, *I* ^2^ = 31%, *n* = 2), but the number of included studies was low. Subgroup analyses for gender showed low heterogeneity for studies with male predominance and unknown gender distributions, and substantial heterogeneity for studies with female predominance or even gender distributions. The pooled S.M.D. was higher for studies that predominately included female participants (S.M.D. = −0.45, 95% CI −0.69 to −0.20, *I* ^2^ = 85%, *n* = 6) compared to studies that predominately included male participants (S.M.D. = −0.18, 95% CI −0.26 to −0.09, *I* ^2^ = 0%, *n* = 2). However, a far smaller number of studies and participants contributed to the male predominance group than to the female predominance group. There was a trend for subgroup differences with regard to study quality.

**Table 3:** Exploratory subgroup analyses for the comparison of fatigue severity in visual impairment vs normal sight

Subgroups	*p*_‐value across subgroups_	Condition	No. of studies	(*n* VI/ control)	S.M.D. (95% CI)	Heterogeneity	
						*I* ^2^	*P*_h_ †
Study quality	0.06	Moderate	5	509/350	−0.18 (−0.40, 0.04)	57%	0.05
		High	9	1991/8045	−0.45 (−0.63, −0.28)	88%	<0.001
Cause of VI	0.73	Other causes of VI	9	1852/6669	−0.35 (−0.54, −0.16)	87%	<0.001
		AMD	3	438/1560	−0.45 (−0.76, −0.14)	82%	0.004
		Other specific eye disorders‡	2	210/166	−0.26 (−0.62, −0.10)	66%	0.09
Study design	0.72	Case‐control	7	1316/1254	−0.32 (−0.61, −0.02)	91%	0.03
		Cross‐sectional	7	1184/7141	−0.37 (−0.50, −0.25)	62%	0.001
Vison loss severity	0.41	VA less than 20/60	5	607/605	−0.44 (−0.84, −0.05)	88%	<0.001
		VA less than 20/40	4	838/5571	−0.43 (−0.62, −0.24)	79%	0.002
		Unknown	3	432/1568	−0.37 (−0.63, −0.12)	74%	0.02
		VA less than 20/200	2	623/651	0.06 (−0.48, 0.59)	77%	0.04
Studied region	<0.001	Asia	5	1167/2066	−0.23 (−0.34, −0.12)	35%	0.19
		North America	3	605/2845	−0.30 (−0.58, −0.03)	83%	0.003
		Australia	2	211/3020	−0.33 (−0.75, 0.09)	83%	<0.001
		Europe	2	338/333	−0.84 (−1.04, −0.64)	31%	0.23
		South America	2	179/131	−0.20 (−0.45, 0.05)	17%	0.17
Socioeconomic region§	0.19	Developed	8	1752/6813	−0.41 (−0.61, −0.20)	90%	<0.001
		Developing	6	748/1582	−0.25 (−0.37, −0.13)	22%	0.27
Gender	0.04	Female predominance (≥60%)	6	888/4757	−0.45 (−0.69, −0.20)	85%	<0.001
		No gender predominance	4	642/1647	−0.25 (−0.58, −0.08)	83%	<0.001
		Male predominance (≥60%)	2	855/1717	−0.18 (−0.26, −0.09)	0%	0.75
		Unknown	2	115/274	−0.48 (−0.74, −0.23)	0%	0.32
Age	0.62	Aged > 65 years	8	1137/5784	−0.34 (−0.50, −0.18)	74%	<0.001
		Aged ≤ 65 years	4	1248/2337	−0.33 (−0.67, 0.02)	94%	<0.001
		Unknown	2	115/274	−0.48 (−0.74, −0.23)	0%	0.32
Defining VA	0.25	Ophthalmic evaluation	8	1298/7241	−0.41 (−0.55, −0.28)	68%	0.003
		Self‐report	2	623/651	0.06 (−0.48, 0.59)	77%	<0.001
		Record linkage	4	579/503	−0.39 (−0.82, 0.04)	91%	0.13

AMD, age‐related macular degeneration; CI, confidence interval; No., number; S.M.D., standardized mean difference; VA, visual acuity; VI, visual impairment.

^†^ *p‐*value of heterogeneity test.

^‡^ One study included patients with diabetic retinopathy and one study included glaucoma patients.

^§^ Socioeconomic status of studied country according to World Economic Situation and Prospects report 2015 of the United Nations.

#### Visual impairment vs normal sight: fatigue odds

**Figure 3:**
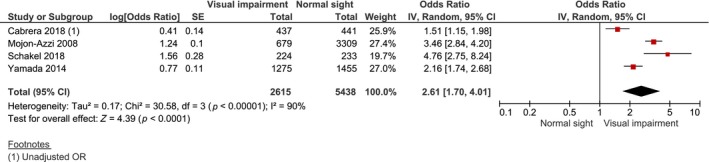
Forest plot of meta‐analysis: multivariable adjusted OR of the presence of fatigue between visually impaired patients and normally sighted controls. OR, odds ratio; S.E., standard error; CI, confidence interval.

A total of four studies that measured the association between visual impairment and fatigue were synthesised by a random effects model in this meta‐analysis, which involved 2615 visually impaired patients and 5438 normally sighted controls (*Figure* 3). The pooled adjusted OR was significant and showed a higher odds of fatigue for visually impaired patients compared with normally sighted controls (OR = 2.61, 95% CI 1.69 to 4.04, *I* ^2^ = 90%, *n* = 4, *Figure* 3). Exclusion of single studies for the purpose of sensitivity analyses had no effect on the statistical significance of the pooled estimate. Funnel plots were not inspected because of the limited number of included studies.

#### Association between fatigue and vision loss severity

**Figure 4:**
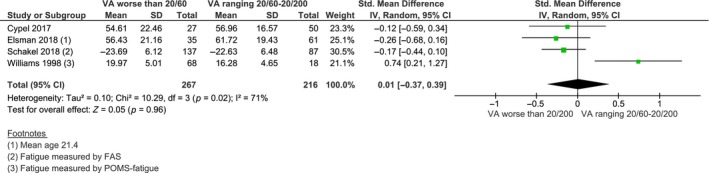
Forest plot of meta‐analysis: S.M.D. of fatigue severity between patients with moderate visual impairment and patients with severe visual impairment or blindness. S.M.D. standardised mean difference, S.D., standard deviation; CI, confidence interval; FAS, Fatigue Assessment Scale; POMS, Profile of Mood States; VA, visual acuity.

Four studies provided sufficient information for our secondary aim to examine the association between fatigue and vision loss severity. Results from the random‐effects meta‐analysis indicated that there was no significant difference in fatigue severity between patients with moderate visual impairment and patients with severe visual impairment or blindness (S.M.D. = 0.01, 95% CI −0.37 to 0.39, *I* ^2^ = 71%, *n* = 4, *Figure* 4). A sensitivity analysis that excluded the study of Williams *et al*. (1998)^42^ reduced heterogeneity to 0% and slightly altered the magnitude and statistical relevance of the pooled estimate. After exclusion, there was a trend towards a small effect for greater fatigue severity in persons with severe visual impairment or blindness compared to persons with moderate visual impairment (S.M.D. = −0.18, 95% CI −0.39 to 0.02, *I* ^2^ = 0%, *n* = 3).

## Discussion

To the best of our knowledge, the present meta‐analysis is the first to compare fatigue levels between patients with visual impairment and normally sighted controls. Based on 14 observational studies of moderate to high quality, we found that fatigue symptoms were more severe in visually impaired adults than in adults with normal sight. This is further supported by results of four normative comparison studies demonstrating that persons with vision loss have worse fatigue symptoms than age‐matched population controls. Both findings are in agreement with the narrative review of Mills *et al*. (2009), who concluded that vitality was the most affected domain of quality of life in patients with glaucoma.^43^ Their claims seem to be somewhat exaggerated however, because they are based on data of observational studies that did not involve comparison groups. The S.M.D. for fatigue severity in the present study was robust in sensitivity analyses that excluded studies of moderate quality and studies that failed to report VA levels, but the effect size was relatively small and not clinically significant. There are several possible explanations for this finding.

First of all, in our meta‐analysis, the effect size might have been underestimated because fatigue was measured by the SF‐36 vitality scale for the majority of the studies. Although sufficient psychometric properties have been reported for fatigue in rheumatoid arthritis^44^ and cancer‐related fatigue,^45^ it is currently unknown whether SF‐36 vitality is a valid and reliable measure of fatigue in patients with visual impairment. Besides, since this subscale was originally developed as a general measure of fatigue, it may lack responsiveness to vision‐specific aspects that have been stressed in our previous qualitative study.^7^ In support of this notion, a meta‐analysis that compared psychological well‐being of persons with visual impairment to sighted peers, found large effect sizes for vision‐specific measures and small effect sizes for generic measures.^46^ Furthermore, several studies have demonstrated that SF‐36 domain scores are only weakly associated with VA or visual field impairments^47^, ^48^ Also for evaluating the impact of low‐vision services and clinical trials, vision‐specific measures are now believed to be more sensitive to the effects of visual impairment than generic measures.^40^, ^49^ For the purpose of our meta‐analysis, however, it was necessary to incorporate a widely used generic measure that permits fatigue comparisons between individuals with visual impairment and those with normal sight.

Another possible explanation for the small effect size of fatigue severity is, that except for Schakel *et al*. (2018),^8^ no studies were specifically aimed at investigating fatigue in relation to visual impairment. Our analyses involved secondary data from observational studies that focused on quality of life of various ophthalmic patient populations. Available data on fatigue was scarce and mostly based on crude values rather than e.g. age‐adjusted estimates. This observation highlights the need for more studies to accurately estimate fatigue severity of patients with visual impairment in comparison to controls with normal sight, in order to support policy makers in allocating resources for research and rehabilitation goals, given the substantial societal costs of comorbid fatigue in this population.^8^

The study showed that visually impaired patients were more than twice as likely to experience fatigue compared to normally sighted controls. This finding, however, should also be interpreted with considerable caution given that only four studies of moderate quality were synthesised in this meta‐analysis. There was a substantial amount of heterogeneity among the studies, possibly due to the various classifications for visual impairment and fatigue assessment tools included. Comparisons were made between glaucoma patients and hospital controls, individuals with visual impairment and normal sight, poor self‐reported general eyesight and excellent self‐reported eyesight, and self‐reported vision and hearing impairment vs no impairment. Furthermore, the inclusion of participants with comorbid hearing impairment in the study of Yamada *et al*. (2014)^32^ may have inflated the observed association with fatigue. However, in the sensitivity analysis, the overall effect was not substantially altered after excluding Yamada *et al*; and even if it is plausible that many older adults may have hearing loss in addition to vision loss,^50^ the presence of hearing impairment was neither measured nor controlled for in the majority of the studies. Nevertheless, the current evidence suggests that patients with visual impairment have a higher odds of experiencing fatigue in comparison with normally sighted individuals.

For our secondary aim, we found a total of six studies that stratified fatigue outcomes for various degrees of vision loss. Because visual impairment categories were defined by slightly different VA values, we pragmatically decided to synthesise findings of four studies with corresponding visual impairment groups. The results suggest that fatigue severity does not differ between patients with moderate visual impairment and patients with severe visual impairment or blindness according to the WHO criteria. This finding may indicate that fatigue cannot be explained by VA alone, but possibly also by compensation efforts and adaptation problems for the indirect consequences of vision loss that have been described before.^7^ However, a trend towards a small effect was observed after removing an outlier study with conflicting results, suggesting that fatigue may be more severe in persons with severe visual impairment or blindness relative to moderate visual impairment. Although based on a small number of studies, this observation is worth noting considering the complete absence of heterogeneity and the overlapping effect estimates and confidence intervals. In contrast to the other studies, Williams *et al*. (1998)^42^ found that persons with legal blindness in both eyes were significantly less fatigued than persons with moderate in the best eye. The authors suggested that the uncertain potential for further vision loss might be more involved in fatigue than what could solely be explained by VA. Taken together, these findings do not give a decisive answer to our second research question. More research is necessary to determine if fatigue in persons with visual impairment is indeed associated with vision loss severity and which psychological adaptation mechanism may play a role in this.

The findings of the present meta‐analysis should be interpreted by its strengths and limitations. Strengths include the elaborate search strategy and the broad inclusion criteria with regard to causes of visual impairment and fatigue outcomes, which enabled us to identify a relatively large amount of studies. To more reliably estimate the association between fatigue and visual impairment, we excluded studies when vision loss was co‐morbid to chronic (inflammatory) conditions that are known for fatigue symptomatology such as multiple sclerosis. Furthermore, our attempts to acquire additional data from corresponding authors enabled us to include two additional studies. Moreover, although based on observational designs, there was a fair amount of high quality studies and no studies of poor quality. Finally, the present study included study populations from various continents including countries in developed‐ and developing regions, which may increase the generalisability of our findings.

Several limitations should be acknowledged as well. First, the findings from the meta‐analyses might have been limited by the substantial level of heterogeneity among various studies. There was great variability between studies with regard to cause of visual impairment, definition of visual impairment, study design and control condition. Exploratory subgroup analyses for fatigue severity suggest that heterogeneity may be explained by the difference in studied region and gender distributions. However, the amount of studies was unequally divided over subgroups and significant heterogeneity remained in some subgroup analyses. Second, the influence of continuous variables such as age were not examined as sources of heterogeneity by meta‐regression in this study. Third, self‐reported measures or certificates for governmental disability benefits, rather than examination of presenting and post‐refraction VA or medical records, were used to establish the diagnosis of visual impairment in some studies. Nevertheless, the consistent results in our subgroup analysis based on vision loss severity and visual impairment definition, together with sensitivity analysis that solely included patients with VA indicative of visual impairment, supported the validity of the association found. Fourth, the stringent inclusion criteria for visual impairment may have limited the number of studies that could be synthesised by meta‐analysis. For example, several studies that investigated specific ophthalmic conditions (such as glaucoma or AMD) were ultimately excluded because VA was either not indicative of visual impairment according to WHO criteria (e.g. mean VA of the study population was better than 20/60 (6/18, 0.33) or cases with unilateral vision loss), or because they failed to report these outcomes. Nevertheless, we believe this methodological decision allows for a more robust estimate of the association between visual impairment and fatigue. Finally, as mentioned before in the discussion, almost all questionnaires that were used in the studies have psychometric properties not specifically tested in a visually impaired sample. More research is necessary to determine whether SF‐36 vitality is a valid measure of fatigue in patients with visual impairment.

## Conclusion

Taken together, our data indicate that visually impaired patients experience higher levels of fatigue severity compared to normally sighted controls. In addition, the presence of visual impairment seems to be associated with an increased odds of fatigue, but more studies of high quality are needed to confirm this finding. Furthermore, the synthesis of available evidence is currently insufficient to support an association between fatigue and vision loss severity. The results of this study provide a better understanding of the magnitude of fatigue severity of patients with visual impairment and have important implications for clinical practice. Ophthalmologists, nurses, optometrists, low vision rehabilitation staff and other health care providers in the field of low vision are advised to discuss fatigue at early stages of treatment and rehabilitation and to closely monitor these symptoms. The development of informative flyers, electronic patient information material or self‐management advices may help raising awareness in a high demanding clinical setting. Future studies are required to clarify how fatigue is associated with visual impairment and to identify underlying mechanisms or important factors involved in this association. Developing interventions which target fatigue for patients with visual impairment should be considered.

## Conflict of interest

The authors report no conflict of interest and have no proprietary interest in any of the materials mentioned in this article.

## Supporting information


**Appendix S1.** Electronic search strategy for bibliographic databases.

**Appendix S2.** Forest plot showing the meta‐analyses for comparisons of fatigue severity levels between visually impaired patients and normally sighted controls, divided by region of studied patient population.
