# Supplementary material for: The association between visual impairment and fatigue: a systematic review and meta-analysis of observational studies
Source: Ophthalmic Physiol Opt. 2019 Nov 6;39(6):399–413. doi: 10.1111/opo.12647 (PMC6899802; doi:10.1111/opo.12647)
Supplement: Supplementary file 1 — Appendix S1. Electronic search strategy for bibliographic databases. [file 44402_2019_3906002_MOESM1_ESM.docx]

**Appendix S1.** Electronic search strategy for bibliographic databases

**PubMed Session Results (03 Apr 2019): 1496 hits**

[Mesh] = Medical subject headings

[Mesh:NoExp] = Medical subject headings without explosion

[tiab] = words in title or abstract or author keywords

| Search |  | Query |
| --- | --- | --- |
| #3 |  | #1 AND #2 |
| #2 |  | "Fatigue"[Mesh] OR "Asthenopia"[Mesh] OR "Asthenia"[Mesh] OR fatigue*[tiab] OR tired[tiab] OR exhaust*[tiab] OR vigor[tiab] OR vigour[tiab] OR sf-36*[tiab] OR Short Form 36*[tiab] OR POMS[tiab] OR vitality[tiab] OR RAND 36*[tiab] OR asthenopia[tiab] OR asthenia[tiab] OR low energy[tiab] OR weary[tiab] OR weariness[tiab] OR wearisome[tiab] OR lassitude[tiab] OR social participation[tiab] |
| #1 |  | "Visually Impaired Persons"[Mesh] OR "Vision Disorders"[Mesh:noexp] OR "Blindness"[Mesh] OR "Vision, Low"[Mesh] OR "Retinal Diseases"[Mesh:noexp] OR "Diabetic Retinopathy"[Mesh] OR "Leber Congenital Amaurosis"[Mesh] OR "Retinal Artery Occlusion"[Mesh] OR "Retinal Degeneration"[Mesh] OR "Retinal Detachment"[Mesh] OR "Retinal Hemorrhage"[Mesh] OR "Retinal Neovascularization"[Mesh] OR "Retinal Vein Occlusion"[Mesh] OR "Vitreoretinopathy, Proliferative"[Mesh] OR "Eye Diseases"[Mesh:noexp] OR "Corneal Diseases"[Mesh] OR "Eye Diseases, Hereditary"[Mesh] OR "Ocular Hypertension"[Mesh] OR "Optic Nerve Diseases"[Mesh] OR "Hemianopsia"[Mesh] OR visually impair*[tiab] OR visual impair*[tiab] OR vision impair*[tiab] OR blindness*[tiab] OR low vision*[tiab] OR reduced vision*[tiab] OR subnormal vision*[tiab] OR diminished vision*[tiab] OR vision disorder*[tiab] OR visual disorder*[tiab] OR vision disab*[tiab] OR visual disab*[tiab] OR visually disab*[tiab] OR retinal disease*[tiab] OR retina disease*[tiab] OR retinal disorder*[tiab] OR retina disorder*[tiab] OR diabetic retinopath*[tiab] OR leber[tiab] OR leber's[tiab] OR lebers[tiab] OR retinal artery occlusion*[tiab] OR retinal degeneration*[tiab] OR retina degeneration*[tiab] OR macular degeneration*[tiab] OR macula degeneration*[tiab] OR macular dystroph*[tiab] OR macula dystroph*[tiab] OR maculopath*[tiab] OR macular edema*[tiab] OR macula edema*[tiab] OR macular oedema*[tiab] OR macula oedema*[tiab] OR retinitis pigmentosa[tiab] OR Rod-Cone dystroph*[tiab] OR Cone-Rod dystroph*[tiab] OR retinal detachment*[tiab] OR retina detachment*[tiab] OR retinal hemorrhage*[tiab] OR retinal haemorrhage*[tiab] OR retinal neovascularization*[tiab] OR retinal neovascularisation*[tiab] OR retinal vein occlusion*[tiab] OR vitreoretinopath*[tiab] OR vitreo-retinopath*[tiab] OR corneal disease*[tiab] OR cornea disease*[tiab] OR corneal disorder*[tiab] OR cornea disorder*[tiab] OR glaucoma*[tiab] OR optic nerve disease*[tiab] OR optic nerve disorder*[tiab] OR optic neuropath*[tiab] OR optic atroph*[tiab] OR hemianop*[tiab] OR quadrantanop*[tiab] OR adynamia[tiab] OR legasthenia[tiab] |

**Embase.com Session Results (03 Apr 2019): 1694 hits**

/exp = EMtree keyword with explosion

/de = EMtree keyword without explosion

:ab,ti,kw = words in title or abstract or author keywords

NEAR/3 = words near to each other, 3 places apart

| Search | Query |
| --- | --- |
| #5 | #3 AND #4 |
| #4 | 'quality of life'/exp OR ((life NEAR/3 qualit*):ab,ti,kw) OR ((living NEAR/3 qualit*):ab,ti,kw) OR 'health status'/exp OR ((health NEAR/3 status):ab,ti,kw) OR ((health NEAR/3 level*):ab,ti,kw) OR qol:ab,ti,kw OR hrql:ab,ti,kw OR hrqol:ab,ti,kw |
| #3 | #1 AND #2 |
| #2 | 'fatigue'/de OR 'exhaustion'/de OR 'lassitude'/de OR 'muscle fatigue'/de OR 'asthenopia'/de OR 'asthenia'/de OR fatigue:ab,ti,kw OR tired:ab,ti,kw OR exhaust*:ab,ti,kw OR vigor:ab,ti,kw OR vigour:ab,ti,kw OR vitality:ab,ti,kw OR 'sf-36':ab,ti,kw OR 'short form*':ab,ti,kw OR poms:ab,ti,kw OR 'rand 36*':ab,ti,kw OR asthenopia:ab,ti,kw OR astenhia:ab,ti,kw OR 'low energy':ab,ti,kw OR weary:ab,ti,kw OR weariness:ab,ti,kw OR wearisome:ab,ti,kw OR lassitude:ab,ti,kw OR 'social participation':ab,ti,kw |
| #1 | 'visually impaired person'/exp OR 'visual disorder'/de OR 'visual impairment'/exp OR 'retina disease'/de OR 'diabetic retinopathy'/exp OR 'retina artery occlusion'/exp OR 'retina degeneration'/exp OR 'retina detachment'/exp OR 'retina hemorrhage'/exp OR 'retina neovascularization'/exp OR 'retina vein occlusion'/exp OR 'vitreoretinopathy'/exp OR 'eye disease'/de OR 'cornea disease'/exp OR 'glaucoma'/exp OR 'intraocular pressure abnormality'/de OR 'optic nerve disease'/exp OR 'hemianopia'/exp OR 'visually impair*':ab,ti,kw OR 'visual impair*':ab,ti,kw OR blindness*:ab,ti,kw OR 'low vision*':ab,ti,kw OR 'reduced vision*':ab,ti,kw OR 'subnormal vision*':ab,ti,kw OR 'diminished vision*':ab,ti,kw OR 'visual disorder*':ab,ti,kw OR 'visually disab*':ab,ti,kw OR 'diabetic retinopath*':ab,ti,kw OR leber:ab,ti,kw OR 'leber s':ab,ti,kw OR lebers:ab,ti,kw OR 'retinal artery occlusion*':ab,ti,kw OR 'retinal degeneration*':ab,ti,kw OR 'retina degeneration*':ab,ti,kw OR 'macular degeneration*':ab,ti,kw OR 'macula degeneration*':ab,ti,kw OR 'macular dystroph*':ab,ti,kw OR 'macula dystroph*':ab,ti,kw OR maculopath*:ab,ti,kw OR 'macular edema*':ab,ti,kw OR 'macula edema*':ab,ti,kw OR 'macular oedema*':ab,ti,kw OR 'macula oedema*':ab,ti,kw OR 'retinitis pigmentosa':ab,ti,kw OR 'rod-cone dystroph*':ab,ti,kw OR 'cone-rod dystroph*':ab,ti,kw OR 'retinal detachment*':ab,ti,kw OR 'retina detachment*':ab,ti,kw OR 'retinal hemorrhage*':ab,ti,kw OR 'retinal haemorrhage*':ab,ti,kw OR 'retinal neovascularization*':ab,ti,kw OR 'retinal neovascularisation*':ab,ti,kw OR 'retinal vein occlusion*':ab,ti,kw OR vitreoretinopath*:ab,ti,kw OR 'vitreo-retinopath*':ab,ti,kw OR 'corneal disease*':ab,ti,kw OR 'cornea disease*':ab,ti,kw OR 'corneal disorder*':ab,ti,kw OR 'cornea disorder*':ab,ti,kw OR glaucoma*:ab,ti,kw OR 'optic nerve disease*':ab,ti,kw OR 'optic nerve disorder*':ab,ti,kw OR 'optic neuropath*':ab,ti,kw OR 'optic atroph*':ab,ti,kw OR ((vision NEAR/3 impair*):ab,ti,kw) OR ((vision NEAR/3 disorder*):ab,ti,kw) OR ((vision NEAR/3 disab*):ab,ti,kw) OR ((visual NEAR/3 disab*):ab,ti,kw) OR ((retina* NEAR/3 disease*):ab,ti,kw) OR ((retina* NEAR/3 disorder*):ab,ti,kw) OR hemianop*:ab,ti,kw OR quadrantanop*:ab,ti,kw OR adynamia:ab,ti,kw OR legasthenia:ab,ti,kw |

**Ebsco / PsycINFO Session Results (03 Apr 2019): 279 hits**

DE = descriptors, thesaurus terms

TI = words in title

AB = words in abstract

KW = author keywords

| Search | Query |
| --- | --- |
| S3 | S1 AND S2 |
| S2 | DE "Fatigue" OR DE "Asthenia" OR TI (fatigue* OR tired OR exhaust* OR vigor OR vigour OR "sf-36*" OR "Short Form 36*" OR POMS OR vitality OR "RAND 36*" OR asthenopia OR asthenia OR "low energy" OR weary OR weariness OR wearisome OR lassitude OR "social participation") OR AB (fatigue* OR tired OR exhaust* OR vigor OR vigour OR "sf-36*" OR "Short Form 36*" OR POMS OR vitality OR "RAND 36*" OR asthenopia OR asthenia OR "low energy" OR weary OR weariness OR wearisome OR lassitude OR "social participation") OR KW (fatigue* OR tired OR exhaust* OR vigor OR vigour OR "sf-36*" OR "Short Form 36*" OR POMS OR vitality OR "RAND 36*" OR asthenopia OR asthenia OR "low energy" OR weary OR weariness OR wearisome OR lassitude OR "social participation") |
| S1 | DE "Partially Sighted" OR DE "Vision Disorders" OR DE "Blind" OR DE "Eye Disorders" OR DE "Refraction Errors" OR DE "Glaucoma" OR DE "Optic Neuritis" OR DE "Hemianopia" OR TI ("visually impair*" OR "visual impair*" OR "vision impair*" OR blindness* OR "low vision*" OR "reduced vision*" OR "subnormal vision*" OR "diminished vision*" OR "vision disorder*" OR "visual disorder*" OR "vision disab*" OR "visual disab*" OR "visually disab*" OR "retinal disease*" OR "retina disease*" OR "retinal disorder*" OR "retina disorder*" OR "diabetic retinopath*" OR leber OR leber's OR lebers OR "retinal artery occlusion*" OR "retinal degeneration*" OR "retina degeneration*" OR "macular degeneration*" OR "macula degeneration*" OR "macular dystroph*" OR "macula dystroph*" OR maculopath* OR "macular edema*" OR "macula edema*" OR "macular oedema*" OR "macula oedema*" OR "retinitis pigmentosa" OR "Rod-Cone dystroph*" OR "Cone-Rod dystroph*" OR "retinal detachment*" OR "retina detachment*" OR "retinal hemorrhage*" OR "retinal haemorrhage*" OR "retinal neovascularization*" OR "retinal neovascularisation*" OR "retinal vein occlusion*" OR vitreoretinopath* OR "vitreo-retinopath*" OR "corneal disease*" OR "cornea disease*" OR "corneal disorder*" OR "cornea disorder*" OR glaucoma* OR "optic nerve disease*" OR "optic nerve disorder*" OR "optic neuropath*" OR "optic atroph*" OR hemianop* OR quadrantanop* OR adynamia OR legasthenia) OR AB ("visually impair*" OR "visual impair*" OR "vision impair*" OR blindness* OR "low vision*" OR "reduced vision*" OR "subnormal vision*" OR "diminished vision*" OR "vision disorder*" OR "visual disorder*" OR "vision disab*" OR "visual disab*" OR "visually disab*" OR "retinal disease*" OR "retina disease*" OR "retinal disorder*" OR "retina disorder*" OR "diabetic retinopath*" OR leber OR leber's OR lebers OR "retinal artery occlusion*" OR "retinal degeneration*" OR "retina degeneration*" OR "macular degeneration*" OR "macula degeneration*" OR "macular dystroph*" OR "macula dystroph*" OR maculopath* OR "macular edema*" OR "macula edema*" OR "macular oedema*" OR "macula oedema*" OR "retinitis pigmentosa" OR "Rod-Cone dystroph*" OR "Cone-Rod dystroph*" OR "retinal detachment*" OR "retina detachment*" OR "retinal hemorrhage*" OR "retinal haemorrhage*" OR "retinal neovascularization*" OR "retinal neovascularisation*" OR "retinal vein occlusion*" OR vitreoretinopath* OR "vitreo-retinopath*" OR "corneal disease*" OR "cornea disease*" OR "corneal disorder*" OR "cornea disorder*" OR glaucoma* OR "optic nerve disease*" OR "optic nerve disorder*" OR "optic neuropath*" OR "optic atroph*" OR hemianop* OR quadrantanop* OR adynamia OR legasthenia) OR KW ("visually impair*" OR "visual impair*" OR "vision impair*" OR blindness* OR "low vision*" OR "reduced vision*" OR "subnormal vision*" OR "diminished vision*" OR "vision disorder*" OR "visual disorder*" OR "vision disab*" OR "visual disab*" OR "visually disab*" OR "retinal disease*" OR "retina disease*" OR "retinal disorder*" OR "retina disorder*" OR "diabetic retinopath*" OR leber OR leber's OR lebers OR "retinal artery occlusion*" OR "retinal degeneration*" OR "retina degeneration*" OR "macular degeneration*" OR "macula degeneration*" OR "macular dystroph*" OR "macula dystroph*" OR maculopath* OR "macular edema*" OR "macula edema*" OR "macular oedema*" OR "macula oedema*" OR "retinitis pigmentosa" OR "Rod-Cone dystroph*" OR "Cone-Rod dystroph*" OR "retinal detachment*" OR "retina detachment*" OR "retinal hemorrhage*" OR "retinal haemorrhage*" OR "retinal neovascularization*" OR "retinal neovascularisation*" OR "retinal vein occlusion*" OR vitreoretinopath* OR "vitreo-retinopath*" OR "corneal disease*" OR "cornea disease*" OR "corneal disorder*" OR "cornea disorder*" OR glaucoma* OR "optic nerve disease*" OR "optic nerve disorder*" OR "optic neuropath*" OR "optic atroph*" OR hemianop* OR quadrantanop* OR adynamia OR legasthenia) |

**Wiley / Cochrane Library Session Results (03 Apr 2019): 1008 hits**

:ab,ti,kw = words in title or abstract or author keywords

| Search | Query |
| --- | --- |
| #3 | #1 AND #2 |
| #2 | (fatigue* or tired or exhaust* or vigor or vigour or (sf NEXT 36*) or (Short NEXT Form NEXT 36*) or POMS or vitality or (RAND NEXT 36*) or asthenopia or asthenia or (low NEXT energy) or weary or weariness or wearisome or lassitude or (social NEXT participation)):ab,ti,kw |
| #1 | ((visually NEXT impair*) or (visual NEXT impair*) or (vision NEXT impair*) or blindness* or (low NEXT vision*) or (reduced NEXT vision*) or (subnormal NEXT vision*) or (diminished NEXT vision*) or (vision NEXT disorder*) or (visual NEXT disorder*) or (vision NEXT disab*) or (visual NEXT disab*) or (visually NEXT disab*) or (retinal NEXT disease*) or (retina NEXT disease*) or (retinal NEXT disorder*) or (retina NEXT disorder*) or (diabetic NEXT retinopath*) or leber or leber's or lebers or (retinal NEXT artery NEXT occlusion*) or (retinal NEXT degeneration*) or (retina NEXT degeneration*) or (macular NEXT degeneration*) or (macula NEXT degeneration*) or (macular NEXT dystroph*) or (macula NEXT dystroph*) or maculopath* or (macular NEXT edema*) or (macula NEXT edema*) or (macular NEXT oedema*) or (macula NEXT oedema*) or (retinitis NEXT pigmentosa) or (Rod-Cone NEXT dystroph*) or (Cone-Rod NEXT dystroph*) or (retinal NEXT detachment*) or (retina NEXT detachment*) or (retinal NEXT hemorrhage*) or (retinal NEXT haemorrhage*) or (retinal NEXT neovascularization*) or (retinal NEXT neovascularisation*) or (retinal NEXT vein NEXT occlusion*) or vitreoretinopath* or (vitreo NEXT retinopath*) or (corneal NEXT disease*) or (cornea NEXT disease*) or (corneal NEXT disorder*) or (cornea NEXT disorder*) or glaucoma* or (optic NEXT nerve NEXT disease*) or (optic NEXT nerve NEXT disorder*) or (optic NEXT neuropath*) or (optic NEXT atroph*) or hemianop* or quadrantanop* or adynamia or legasthenia):ab,ti,kw |
