# Supplementary material for: The association between visual impairment and fatigue: a systematic review and meta-analysis of observational studies
Source: Ophthalmic Physiol Opt. 2019 Nov 6;39(6):399–413. doi: 10.1111/opo.12647 (PMC6899802; doi:10.1111/opo.12647)
Supplement: Supplementary file 2 — Appendix S2. Forest plot showing the meta-analyses for comparisons of fatigue severity levels between visually impaired patients and normally sighted controls, divided by region of studied patient population. [file 44402_2019_3906002_MOESM2_ESM.docx]

**Appendix S2.** Forest plot showing the meta-analyses for comparisons of fatigue severity levels between visually impaired patients and normally sighted controls, divided by region of studied patient population.


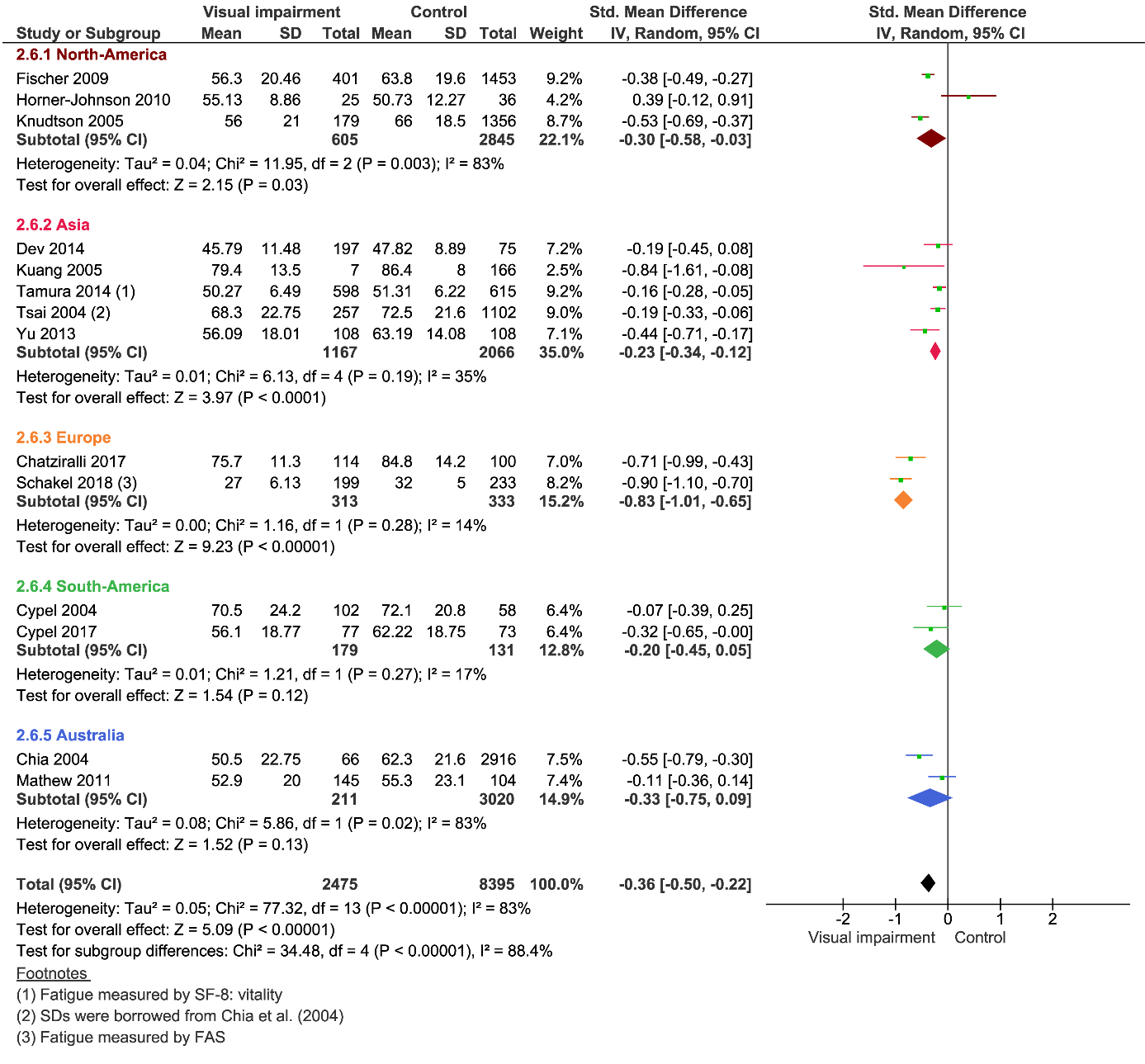


SMD standardized mean difference, SD standard deviation, CI confidence interval, FAS Fatigue Assessment Scale, SF-8 Medical Outcomes Study Short-Form 8 questionnaire.
